# Hidden Markov Models reveal a clear human footprint on the movements of highly mobile African wild dogs

**DOI:** 10.1038/s41598-020-74329-w

**Published:** 2020-10-21

**Authors:** Scott Creel, Johnathan Merkle, Thandiwe Mweetwa, Matthew S. Becker, Henry Mwape, Twakundine Simpamba, Chuma Simukonda

**Affiliations:** 1 Zambian Carnivore Programme, P.O. Box 80, Mfuwe, Eastern Province Zambia; 2 Department of Ecology, Montana State University, Bozeman, MT 59717 USA; 3 Institut för Vilt, Fisk Och Miljö, Sveriges lantbruksuniversitet, Umeå, Sweden; 4 Zambia Department of National Parks and Wildlife, Chilanga, Zambia

**Keywords:** Ecology, Environmental sciences

## Abstract

Large carnivores have experienced considerable range contraction, increasing the importance of movement across human-altered landscapes between small, isolated populations. African wild dogs (*Lycaon pictus*) are exceptionally wide-ranging, and recolonization is an important element of their persistence at broad scales. The competition-movement-connection hypothesis suggests that adaptations to move through areas that are unfavorable due to dominant competitors might promote the ability of subordinate competitors (like wild dogs) to move through areas that are unfavorable due to humans. Here, we used hidden Markov models to test how wild dog movements were affected by the Human Footprint Index in areas inside and outside of South Luangwa National Park. Movements were faster and more directed when outside the National Park, but slowed where the human footprint was stronger. Our results can be directly and quantitatively applied to connectivity planning, and we use them to identify ways to better understand differences between species in recent loss of connectivity.

## Introduction

Current extinction rates are unprecedented in human history^1^, and large mammals are among the most affected taxa^2^. Large carnivores have experienced particularly large declines in numbers and geographic distribution, first in Europe and North America and now on other continents including Africa^3,4^. Many African ecosystems have maintained all of their large carnivores, but the lion (*Panthera leo*), cheetah (*Acinonyx jubatus*) and African wild dog (*Lycaon pictus*) are all considered vulnerable or endangered, and all show decreasing population trends^5–7^. Understanding and promoting the processes that allow demographic and genetic connections between increasingly small and isolated populations of such species is critical for their long-term conservation. Without connectivity, local extirpations are more likely, genetic variability erodes and species are deprived of adaptive potential.

Research on connectivity grew out of biogeography, and much of this work has focused on testing how the area and isolation of patches affects connectivity and metapopulation dynamics^8–10^. However, a comprehensive review of data from 1015 species on six continents found that “patch area and isolation are surprisingly poor predictors of occupancy for most species”^11^, and as summarized by Kareiva “it is doubtful whether generalizations can be made about the effects of habitat patchiness [on connectivity] without detailed behavioural studies”^12^. While the importance of species’ traits in mediating the effects of habitat patchiness or fragmentation is well recognized, there is considerable scope to develop our theoretical and empirical understanding of relationships between species traits, movement patterns and connectivity^13^.

Within the African large carnivore guild, the persistence of subordinate competitors (wild dogs and cheetahs) is strongly linked to their ability to detect and avoid unfavorable ecological conditions while locating favorable conditions^14–21^. The competition-movement-connection (CMC) hypothesis suggests that selection for the ability to traverse areas made unfavorable by the presence of dominant competitors may also promote the ability to traverse areas made unfavorable by humans^22^. Fundamentally, the CMC hypothesis simply proposes that decisions about the speed and linearity of movement that evolved in response to competitors also affect responses to anthropogenic changes^23–27^. All that is required is that changes in movement patterns in response to conditions perceived to be unfavorable or dangerous are generalized. If so, fugitive species like the wild dog and cheetah should be better adapted to maintain connectivity in a landscape with high resistance due to anthropogenic effects, relative to dominant competitors like lions and spotted hyenas (*Crocuta crocuta*)^22^.

In addition to effects on individual movement, competition within the carnivore guild affects population density: spotted hyenas and lions outnumber wild dogs and cheetahs in all relatively intact ecosystems studied to date^15–17^. Because low population density reduces gene flow even in the absence of impediments to individual movement^28^, these differences in population density predict a pattern of connectivity opposite to that predicted by the CMC hypothesis. Larger populations also experience less genetic drift, reinforcing the prediction of the competition-density-connection (CDC) hypothesis that the higher densities of dominant competitors should increase genetic connectivity between ecosystems, relative to subordinate competitors^22^.

African wild dogs (*Lycaon pictus*) have long been renowned for their large home ranges^17,29^ and ability to move long distances^30^. Early field studies suggested that wild dogs were nomadic^31^ because (with the technology of the time) they often disappeared for extended periods. Further study revealed that wild dogs have a system of space use similar to most other social carnivores, with territorial defense in the central portion of a home range with well-defined borders. What is unique about wild dogs is the spatial scale of this pattern, with home ranges typically spanning several hundred square kilometers: annual home range size for populations in six ecosystems was 545 ± 49 km^2^ ($\stackrel{-}{X }\pm \mathrm{SEM}$)^17^. As would be expected with home ranges this large, wild dogs make notably large movements on all time scales^29–35^.

Such movements promote the ability to recolonize ecosystems following local extirpation (or to demographically rescue populations that have grown perilously small) which is an important facet of wild dog persistence at national and continental scales^36^. Large carnivores are well-represented in studies of the ways that human modification of the landscape reduces connectivity between ecosystems^37–40^, and recent research has begun to examine this question for wild dogs and other African large carnivores^22,35,41–43^. For wild dogs, Cozzi et al*.* used data from GPS collars to test whether contact with roads or human settlements caused dispersing animals to retreat, i.e. change bearing by > 90° within 24 h, and found that they did so in 24 of 27 cases in which they approached within one kilometer of a village, cattle post or crop field. Cozzi et al*.* did not test whether this value differed from the frequency of such turns in data without human encounters. Rather, they assumed that “[by] chance alone, on 50% of occasions dispersers may have retreated irrespective of human presence”, relying on an implicit assumption that turning angles are uniformly distributed (which they are not: see “Results”). Nonetheless, their results suggest that wild dogs avoid encounters with humans during long distance movements between protected areas. In a separate study in the same area, Cozzi et al. found that a fence designed to separate African buffalo (*Syncerus caffer*) from cattle (*Bos taurus*) had little effect on wild dog movements^44^.

Creel et al. tested how landscape resistance due to the human footprint affected connectivity between three ecosystems for wild dogs in Eastern (Luangwa Valley), Central (Kafue) and Western (Liuwa Plains) Zambia, using single nucleotide polymorphisms (SNPs) at 2584 loci. As would be expected from the movements described by Cozzi et al., Creel et al. found little differentiation between ecosystems for wild dogs, and that a model of isolation by resistance^45^ due to humans fit the genetic data no better than a model of isolation by distance. These results contrasted sharply with data from SNP genotypes of sympatric lions, which differed considerably between ecosystems and revealed a strong signature of isolation by resistance due to humans^22^.

**Figure 1:**
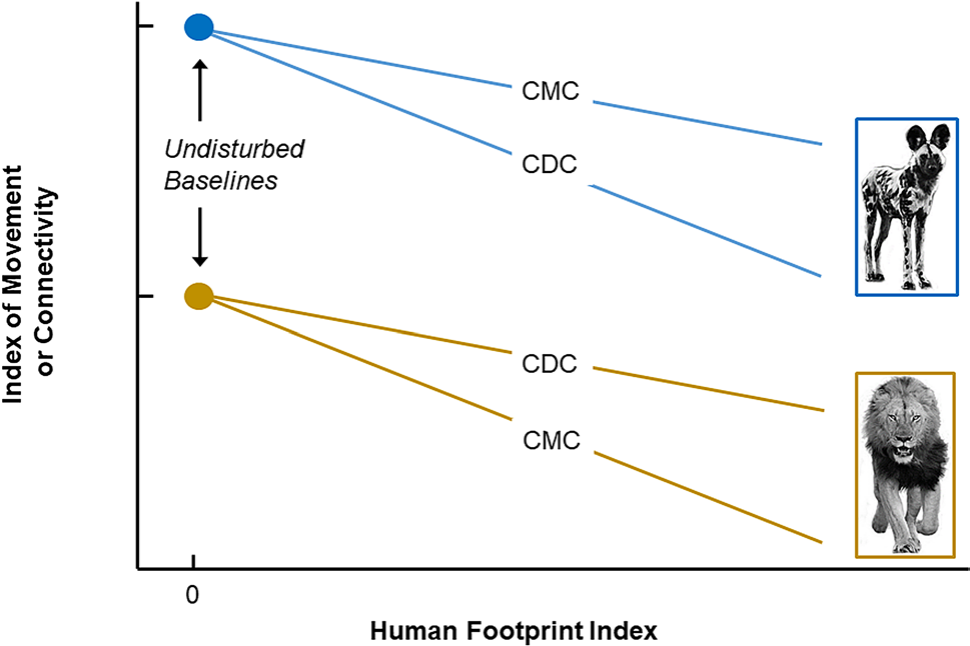
A conceptual diagram of plausible differences between species in anthropogenic effects on connectivity. Genetic data show that connectivity between ecosystems has been stronger for African wild dogs (subordinate competitors) than for lions (dominant competitors). However, it is not yet clear whether this difference exists because the adaptations of wild dogs better equip them to move through areas made unfavorable by humans (as proposed by the competition–movement–connection hypothesis, CMC), or because wild dogs had better past connectivity under undisturbed conditions, now strongly reduced by small population size (as proposed by the competition–density–connection hypothesis, CDC). Studies to date have not described the slopes of these relationships in a manner that allows direct quantitative comparison.

The inferences to be drawn from these studies are not completely clear. On the one hand, genetic data have shown wild dogs are spatially well-connected relative to sympatric large carnivores, but have not shown that the movements that caused these genetic patterns remain common. Data from GPS collars have shown that wild dogs sometimes make long distance movements between protected areas in areas with relatively sparse human settlement, but have also suggested that these movements are affected by avoidance of encounters with humans^32,43,46^. Ambiguities also exist in more detailed patterns. For example, Cozzi et al. reported that wild dogs never crossed two major roads in the southern portion of the Kavango–Zambezi Transfrontier Conservation Area, but wild dogs regularly cross, travel on, and even hunt along the M9 highway in the northern portion of the same TFCA, even though the M9 is Zambia’s primary east–west highway and thus has heavy traffic (and an appreciable number of dogs are killed by collisions)^47^. Abrahms et al. studied wild dogs in the Moremi population from which the dispersers studied by Cozzi et al. originated, and reported that they were attracted to lightly-trafficked dirt roads when travelling, but avoided roads when resting^48^. In short, it is not clear whether (a) the movement capability of wild dogs continues to provide good connectivity even on landscapes that have been modified by humans, or (b) human modification of the landscape reduces connectivity below a high natural baseline (Fig. 1). If the latter is true for a species with the movement capacity of the wild dog, it is likely to be true for most terrestrial vertebrates.

**Figure 2:**
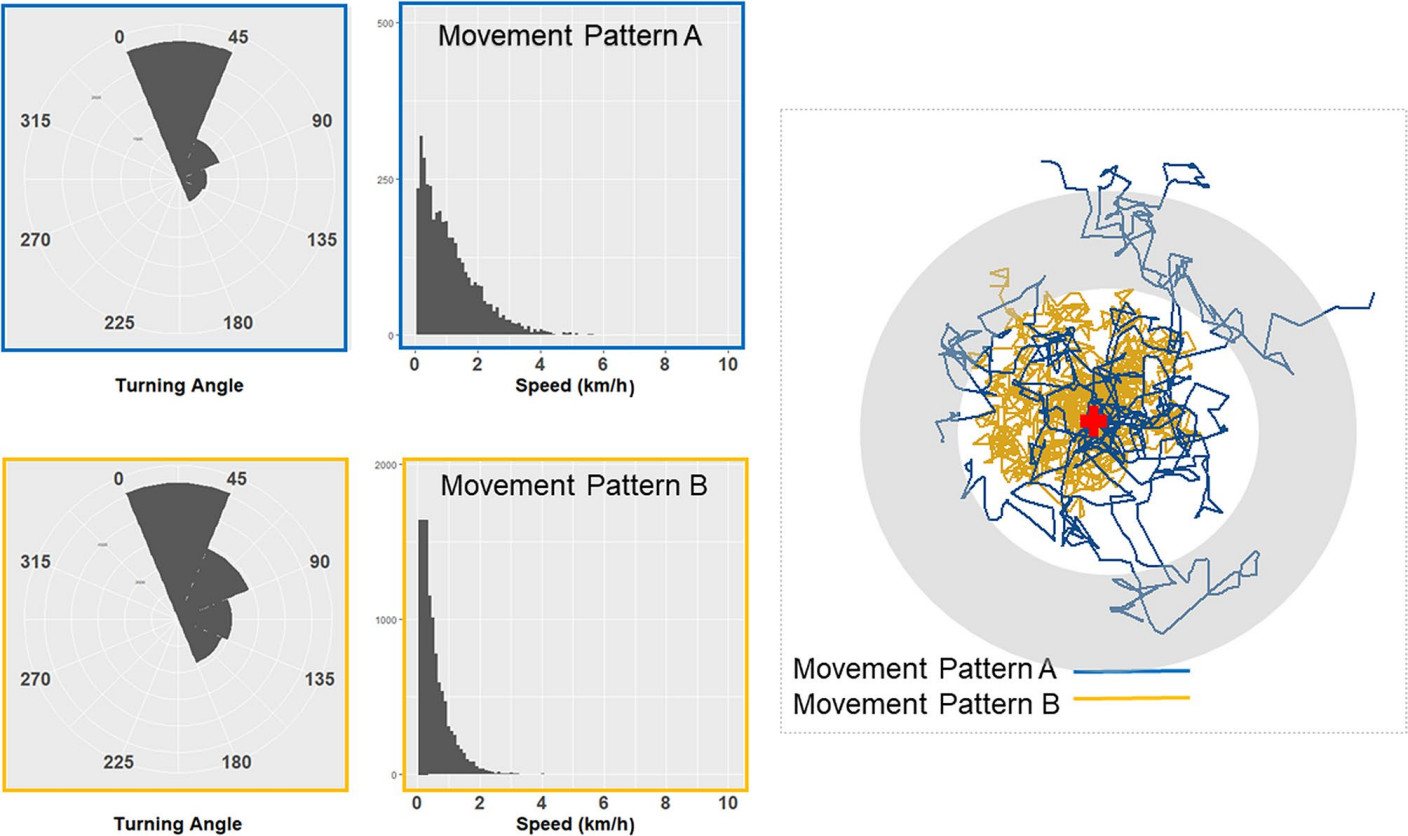
A simple simulation demonstrating that differences in movement speed (i.e., distance travelled in a fixed time period) and turning angle are fundamental drivers of differences in connectivity. At left are the distributions of turning angles and speeds for two movement patterns (**A**: blue, **B**: yellow). At right are individual movement trajectories simulated over 100 time steps by random draws for each of the two patterns, with all individuals starting from the center point (red cross). Movement pattern A, with faster movements (i.e., longer steps) and a lower probability of making sharp turns, produces a higher probability of connecting to distant locations identified by the shaded outer ring.

Here, we used a large and temporally fine-scaled dataset on the movements of wild dogs in Zambia’s Luangwa Valley to test whether patterns of movement were detectably affected by human modification of the landscape. To quantify anthropogenic landscape resistance, we used the Human Footprint Index^49,50^, which aggregates eight types of human land use that are likely to be pertinent for large carnivores (built environments, crop lands, pasture lands, human population density, night lights, railways, roadways, and navigable waterways) and has been shown to explain reductions in movement for many species^51^. To quantify effects on movement, we used hidden Markov models (HMMs), which identify latent states that can be distinguished by movement speed and turning angles (and often have clear interpretations, for example, travelling vs. foraging^52^). HMMs estimate transition probabilities between these states while accounting for serial autocorrelation, and allow direct tests of the effect of environmental factors on the probability of each state^52–56^. Differences between states in speed and turning angle lead to differences in connectivity, as can be shown with a simple simulation. Figure 2 shows data for two patterns of movement that differ in speed and turning angles. Pattern A is characterized by faster movements and shallow (< 45°) turning angles. Pattern B is characterized by slower movements with a higher probability of sharp turns. Simulating movement over 100 time steps with these distributions (Fig. 2), individuals with pattern A commonly connect to distant locations, while individuals with pattern B rarely do so. By quantifying effects on basic attributes of movement that increase or decrease connectivity, HMMs provide a means of examining connectivity in a mechanistic manner (complementing genetic approaches that test for effects on the patterns that these mechanisms produce). The Luangwa Valley Ecosystem is ideal to apply these methods because wild dogs regularly move in areas with low Human Footprint Index values inside South Luangwa National Park and areas with much higher Human Footprint Index values in adjacent buffer zones.

## Methods

### Study site

**Figure 3:**
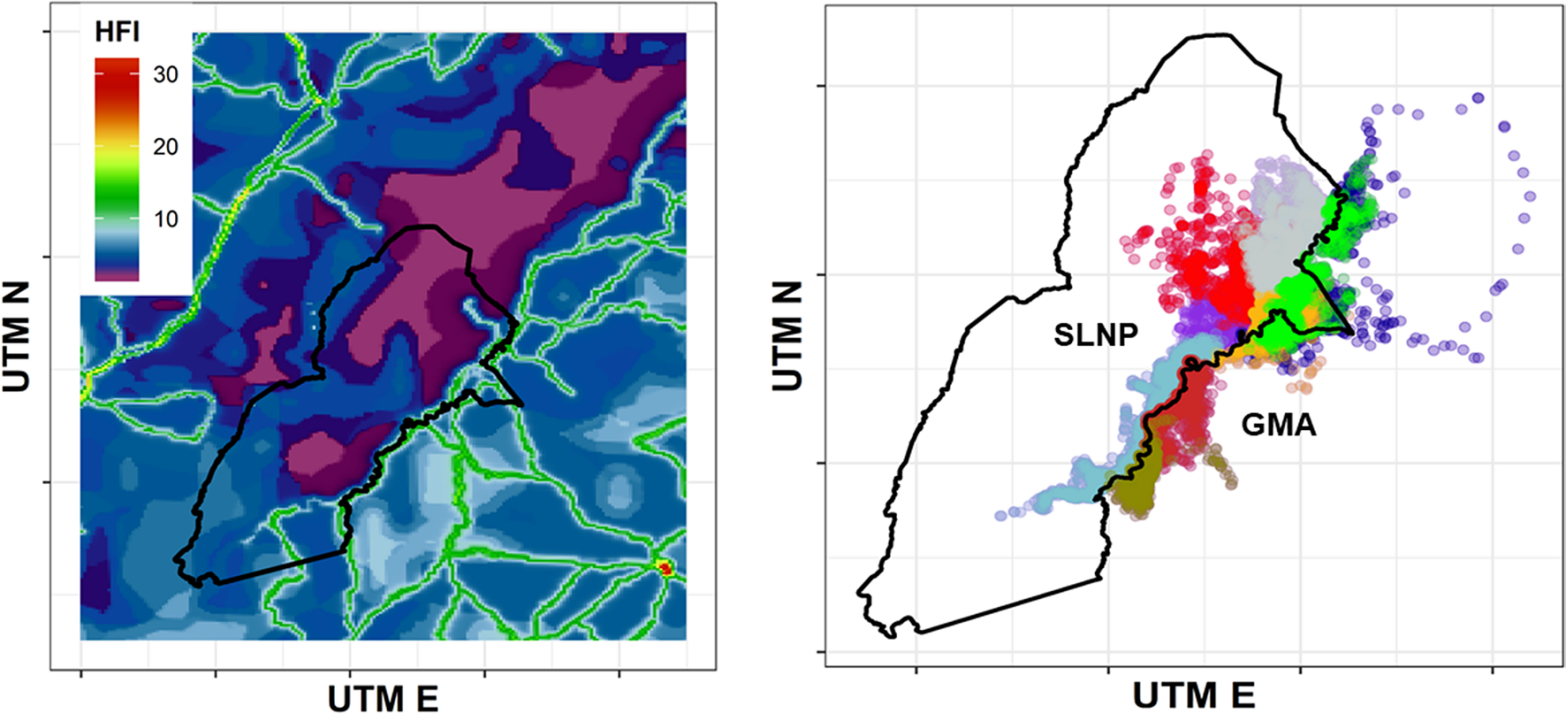
(**A**) Human Footprint Index values mapped for the Luangwa Valley Ecosystem. The boundary of South Luangwa National Park is shown in black. Adjacent areas to the east with a stronger human footprint are in the Lupande and Lumimba Game Management Areas. Values are heat mapped so that violet indicates low HFI and red indicates high HFI. (**B**) 15,185 locations for steps of 80 min duration for nine African wild dogs, to which we fit hidden Markov Models.

The data analyzed here were collected between August 2016 and January 2020 as part of a long-term study of African wild dogs in the Luangwa Valley Ecosystem^47^. Our ~ 5000 km^2^ intensive study area is located along the eastern boundary of South Luangwa National Park and the adjoining Lupande and Lumimba Game Management Areas (GMAs). The ecosystem supports the largest wild dog population in Zambia^47,57^, which is of continental importance. South Luangwa National Park is strictly protected, and the adjacent GMAs are IUCN Category VI buffer zones^58^. The wild dog population in the Luangwa Valley Ecosystem is ideal for this study because it is divided by the Luangwa River flowing from North to South, with high levels of protection and low values of the Human Footprint Index within South Luangwa NP, and an ecologically similar area in the GMAs to the east with lower levels of protection, including areas of cropland, roads, settlement and villages. Individual wild dogs can cross the Luangwa River, particularly during the dry season, and some packs have home ranges entirely within the NP, some entirely within the GMAs, and a few spanning both sides (Fig. 3). Data from this site provided substantial (eightfold) variation in Human Footprint Index values at the locations used by wild dogs (Fig. 3), ranging from a minimum of 2 to a maximum of 16.

Ecologically, both the National Park and the GMAs are a mosaic of edaphic grassland, deciduous riparian forest, miombo (*Brachystegia*) woodland, mopane (*Colophospermum mopane*) woodland and dry deciduous forest^59^. The site experiences a rainy season between December and April and a dry season between May and November. Populations of carnivores and large herbivores concentrate along the Luangwa River at the boundary of the national park and the GMAs, particularly during the dry season^60–62^.

### Radio collar location data

We used direct observation aided by VHF (Telonics MOD-335-3), and GPS (Telonics TGW-4200-2) radiotelemetry to monitor wild dogs in the intensive study area, which at the end of this study numbered 181 recognized individuals. Both of these collars weigh less than half of suggested values for animals of the wild dog’s size from the American Society of Mammalogists, which are the guidelines recognized for IACUC review for US federal research funding. We radicollared wild dogs by intramuscular injection of a combination of medetomidine and zoletil, reversing the medetomidine by intramuscular injection of atipamezole after 45 min to one hour. Anaesthetic drugs were delivered by darting with an air-powered DanInject rifle, with all procedures performed by an experienced and Zambian-registered veterinarian, in collaboration with the Zambia Department of National Parks and Wildlife, with a protocol approved by the MSU IACUC (approval number 2020-123). Darting was restricted to daylight hours and groups that were at rest. When temperatures were hot, darted dogs were gently carried to the nearest shade for collaring and recovery. Only adults were radiocollared. To examine fine-scaled data that meet the assumptions of hidden Markov models and to restrict the analysis to periods of movement, the data were filtered to 15,181 locations with a step duration of 80 min and a minimum step length of 0.2 km, providing a mean of 1688 locations from each of nine packs that were well distributed across the study area (Fig. 3: minimum 907 locations/pack, maximum 2601 locations/pack). Constant time intervals between locations are required to fit HMMs, and 80 min was the shortest interval for which we had a large sample size. African wild dogs rarely move in the middle of the day, other than small movements to remain in shade^17^. To avoid inflating our sample with a large number of repeated locations at resting sites, we filtered steps shorter than 0.2 km, based on a preliminary inspection of the frequency distribution of step lengths (though the exact value of 0.2 was not critical) and treated them as missing values in the time series.

### Hidden Markov modelling

We used these data to fit hidden Markov models (HMMs) in R using the moveHMM package^54^. Model selection using information theory strongly favored an HMM with three movement states over a model with two states (ΔAIC = 952.7). An HMM with four states provided a further reduction in AIC score (ΔAIC = 39.0), but yielded considerable overlap between states that complicated interpretation without altering inferences about the effects of covariates. Thus we based our inferences on the three state HMM, and assessed goodness of fit for the three state model with a QQ plot of pseudoresiduals (obtained with the moveHMM::pr() function) vs fitted values, which showed a good fit (Fig. S1). We modeled step lengths using a gamma distribution, and turning angles using a von Mises distribution^56^ and confirmed that model estimates were not altered by changes in the initial values used for model fitting^54^. We used the moveHMM:viterbi() function to assign the movement state of each individual at each time step, and the moveHMM::sp() function to determine the probabilities of assigned states. This process confirmed that state assignments yielded a mean probability of 87% (SD = 15.5%) for the assigned state.

A priori, we selected three covariates to test for effects on transition probabilities between the three movement states, reflecting our focus on hypothesis testing. First, we included a categorical seasonal covariate that distinguished between denning (Julian dates 160 to 220) and non-denning periods. Newborn wild dog pups cannot follow their pack while it hunts, so the pack returns to a fixed den site after each hunt for approximately two months after pups are born, unlike other times of the year^17^. The other two covariates directly tested anthropogenic effects on movement. One was categorical, distinguishing locations within South Luangwa National Park (*N* = 10,228) from locations in the Game Management Areas (*N* = 4934). This variable accounts for differences in human activities and their consequences that do not directly alter the landscape (e.g., hunting, prey depletion^61,62^). One individual made a long-distance foray from South Luangwa NP though Lumimba GMA into Luambe, Lukusuzi and North Luangwa National Parks (blue fixes to the northeast in Fig. 3), and 19 fixes from that movement were also categorized as within a National Park. The last covariate was the Human Footprint Index^50^, which provides ground-truthed 1 km^2^ resolution mapping of built environments, crop lands, pasture lands, human population density, night lights, railways, roadways, and navigable waterways. The HFI has been shown to detect human effects on the movements of many species, and because it is publicly available for the entire terrestrial surface of the earth, it allows direct, quantitatively comparable analysis between studies. We downloaded the most recent (2009) HFI data made available by Venter et al. from Dryad at https://doi.org/10.5061/dryad.052q5. Within our study area, we confirmed that these HFI values aligned well with the spatial distributions of roads, settlements, human encroachment^63^, illegal harvest using wire snares^64^, and low densities of the large herbivores (impala [*Aepyceros melampus*] & puku [*Kobus vardonii*]) that are wild dogs’ primary prey in this ecosystem^62^. Following Michelot et al. we standardized HFI values prior to fitting HMMs. Covariate values were based on the location at the beginning of a time step.

Having fit a three state HMM with these three covariates as additive effects, we used the moveHMM:plotStationary() function to determine 95% confidence intervals for the effect of each covariate on the stationary probability of each movement state^54,56^.

### Preliminary evaluation of temporal changes in human density and distribution

The most recent HFI dataset^50^ provides values for 2009, and our data for African wild dog movements were collected between 2016 and 2020. This time lag creates the possibility that the spatial distribution of human effects might have changed enough to weaken the HFI as a descriptor. As mentioned above, we confirmed that the HFI map for our study area aligned well with roads, settlements, protected area boundaries and areas with land conversion, reducing this concern. Nonetheless, we tested for changes through time in the spatial distribution of human effects on our study site by downloading 1 km^2^ resolution human population counts for Zambia from the WorldPop project (https://www.worldpop.org/geodata/listing?id=74). These population estimates can also be extracted from the Google Earth Engine, and disaggregate national census data using covariates that include land cover, roads, nightlights, vegetation and topography^65^. These WorldPop estimates of population density are available for every year from 2000 to 2020 (though the same census data underlie estimates for multiple years). We downloaded datasets for 2009, 2016, 2018 & 2020 (i.e., the most recent year for which HFI data were available, and the beginning, mid-point and end of our movement data) and extracted the values at the 15,181 wild dog locations described above.

**Figure 4:**
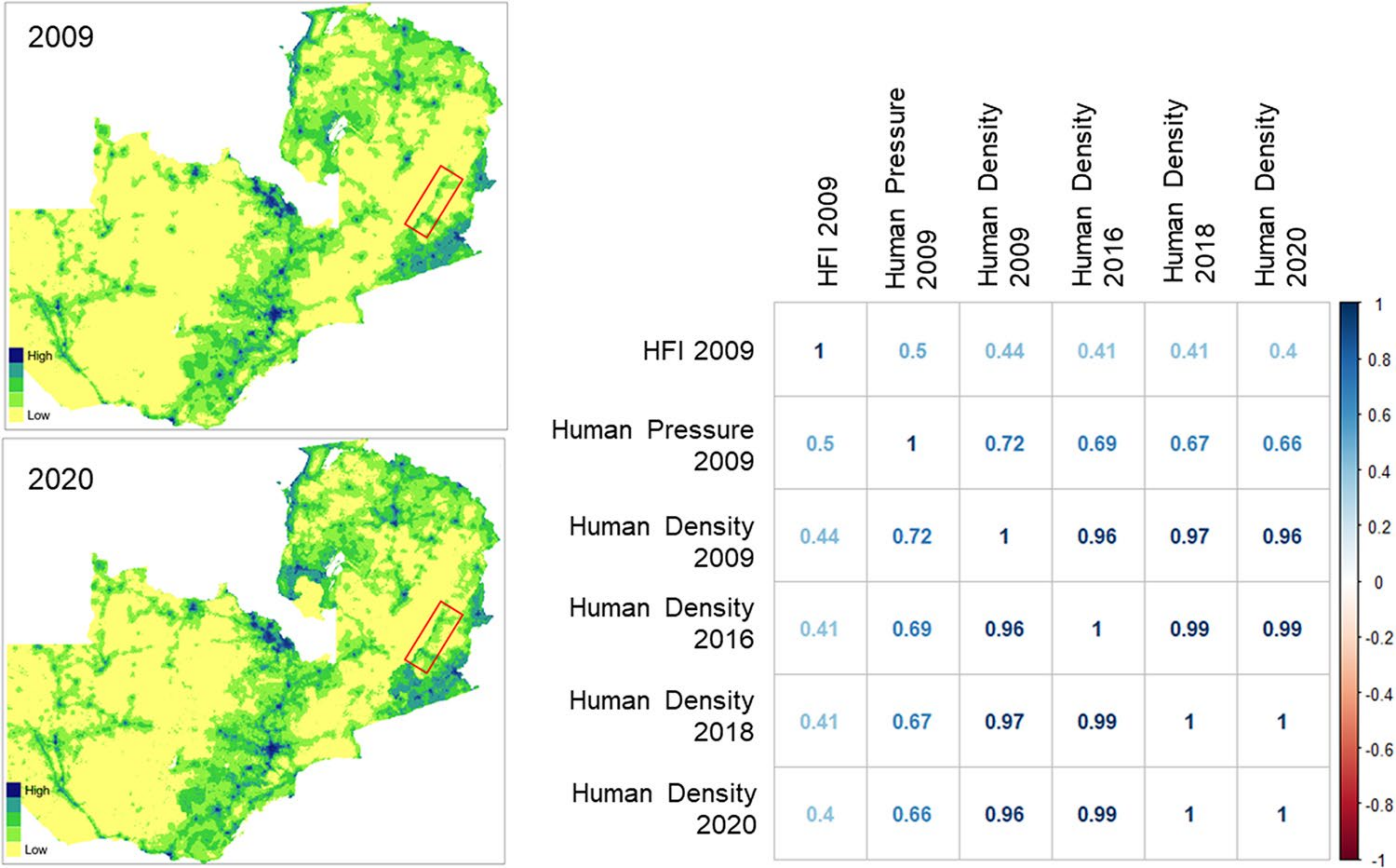
(Left) The spatial distributions of estimated human population density in 2009 and 2020 from the WorldPop dataset for Zambia. The Luangwa Valley is identified by the red rectangle. (Right) Correlations between WorldPop estimates of human density in 2009, 2016, 2018 and 2020 (for the set of locations used by African wild dogs in this study) all fell between 0.96 and 1.00, confirming that the spatial distribution of the human population was relatively constant for this area in this period. Because the Human Footprint Index integrates seven variables in addition to human density, the correlation between WorldPop density estimates and the HFI was lower (0.44) when compared for the same year (2009, the most recent year for which both datasets are available). Differences in methodology cause the correlation between WorldPop estimates and the ‘human population pressure score’ component of the HFI to be intermediate (0.72) when compared for the same year.

We then tested whether the spatial distribution of human effects changed appreciably between 2009 and 2020 at the locations used by wild dogs. Pairwise correlations between WorldPop estimates for 2009, 2016, 2018 and 2020 all fell between 0.96 and 1.00 (Fig. 4), confirming that the spatial distribution was relatively constant over the period of interest. Maps of WorldPop estimates for 2009 and 2020 also confirmed that as estimated human density increased over this period, the spatial distribution of high and low values remained relatively constant (Fig. 4).

## Results

Wild dogs were more than twice as likely (10,228 vs. 4934 locations) to be within the National Park rather than the adjacent GMAs, and the HFI was lower in the National Park (6.39 ± 0.03, $\overline{\overline{X}} \pm SEM$ here and throughout) than it was in the GMAs (8.73 ± 0.04) (Fig. 3). Wild dogs were known to cross the Luangwa River twelve times (in a thirteenth case a single male was killed by crocodiles while attempting to cross).

**Figure 5:**
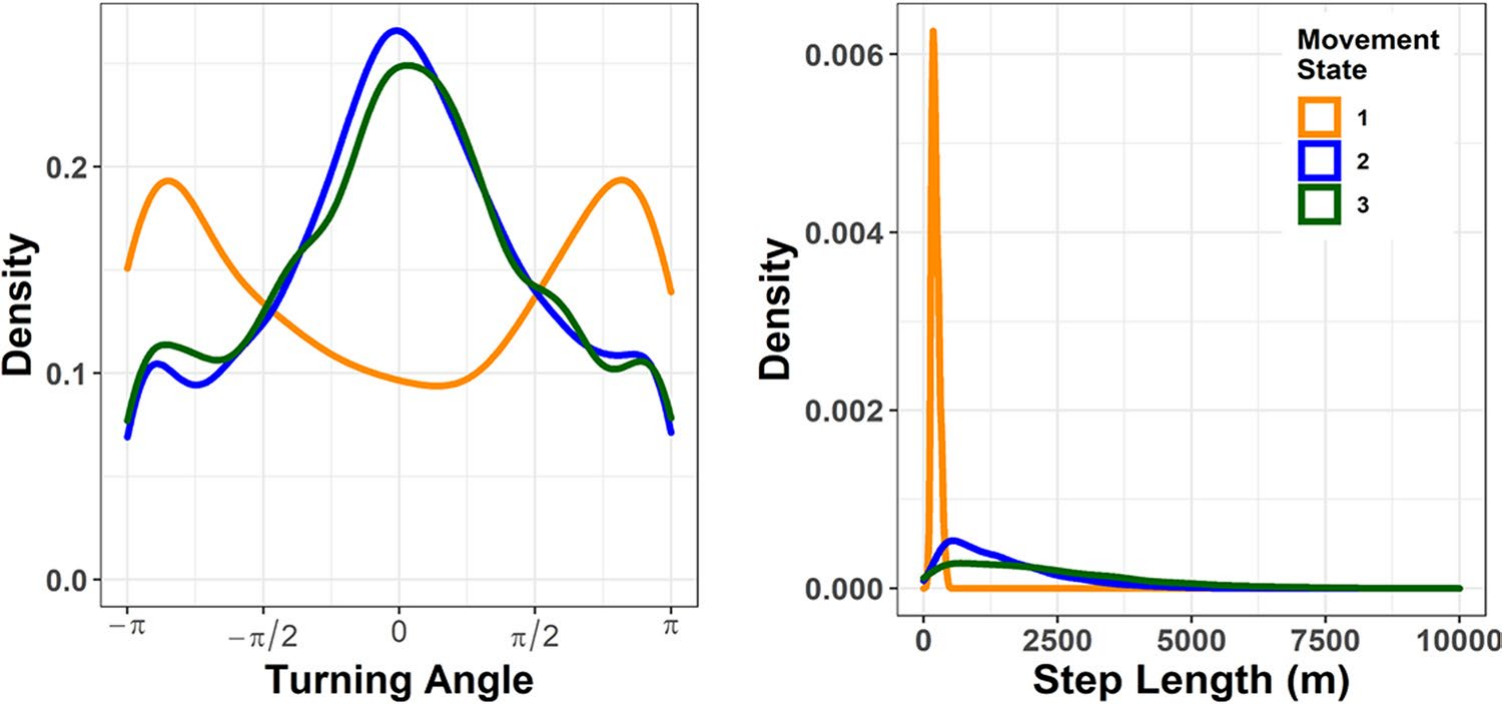
A hidden Markov model with three latent states fit the data well (see Fig. S1). (**A**) The distributions of turning angles for the three movement states. (**B**) The distributions of step lengths for the three movement states. Color coding of states is consistent in Figs. 6, 7.

A three state hidden Markov model provided a good fit (Fig. S1) to data on wild dog movements (having excluded data from periods when they were essentially stationary, as described in the Methods). In this model, State 1 (‘slow’) represents slow movement with a wide range of turning angles, State 2 (‘typical’) represents directed movement at moderate speed and State 3 (‘fast’) represents directed movement at higher speed. With these attributes, the three states represent low (State 1), intermediate (State 2) and high (State 3) spatial displacement over a fixed time period (Fig. 5, Table 1).

**Table 1:** Movement speeds and turning angles for movement states of African wild dogs in the Luangwa Valley Ecosystem from a three state hidden Markov Model.

State^a^	Speed (km/h)		Angle (radians)		*N*
	Mean	Std. Dev	Mean	Std. Dev	
1	0.17	0.05	− 0.036	2.16	1088
2	1.10	0.79	0.057	1.59	9629
3	1.82	1.79	0.024	1.65	4458

^a^Movement states from the decoded state sequence.

**Figure 6:**
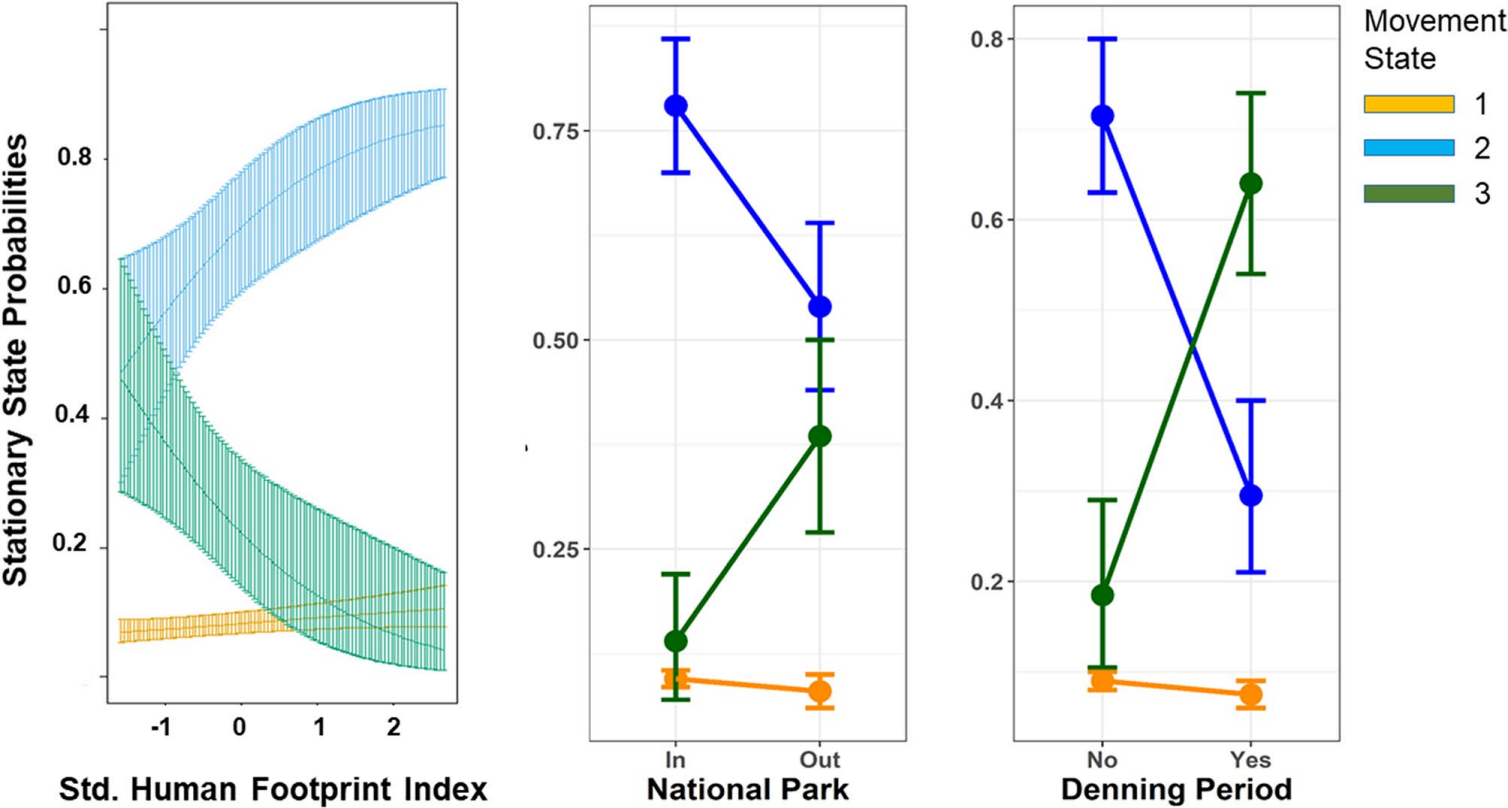
The effects of covariates on the stationary probability of each movement state (1 = slow & meandering, 2 = intermediate speed and directed, 3 = fast and directed). (**A**) Increased human footprint index values strongly reduced the likelihood of fast, directed movement. (**B**) Fast, directed movement was more likely for wild dogs outside the National Park in Game Management Areas. (**C**) Fast, directed movement was more likely during denning periods. Color coding of states is consistent with Figs. 5 and 7.

**Figure 7:**
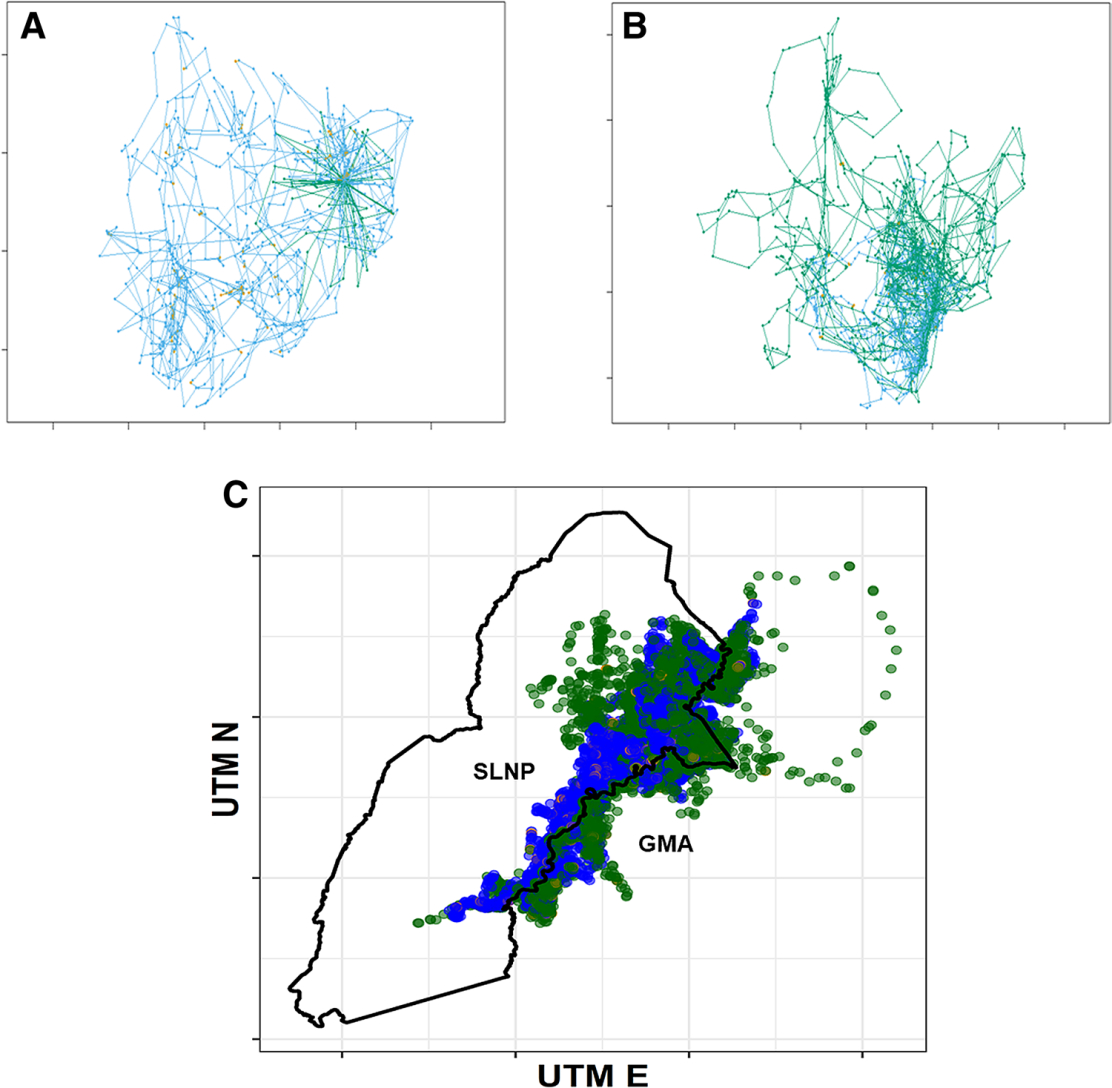
Movements and their locations color coded by their movement state. (**A**) The movements of a single individual to illustrate an increase in the likelihood State 3 movements (green) during a denning period, resulting in an obvious rosette centered on the den location. (**B**) The movements of a second individual to illustrate an increase in the likelihood of State 3 movements (green) during exploratory movements to the north of the pack’s established home range immediately after the death of the alpha female. (**C**) All analyzed locations for all individuals. Slow, undirected movements (state 1) were sufficiently uncommon that they are almost completely masked by states 2 and 3 when all the data are co-plotted. State 3 (fast, directed movement) was significantly more likely for wild dogs in Game Management Areas outside of the National Park (black line) than for wild dogs inside the park. Color coding of states is consistent with Figs. 5 and 6.

All three covariates had strong effects on movement (Fig. 6). During denning periods, wild dogs reduced typical movements (State 2) and increased fast movements (State 3), in response to the necessity to hunt repeatedly from the same location and return to the pups after each hunt. This pattern was readily visible as a ‘rosette’ of State 3 movements centered on a den site, as illustrated in Fig. 7A, where a den site is readily apparent in the eastern portion of movements by one pack. Fast, State 3 movements were also associated with exploratory forays away from established home range cores, as illustrated in Fig. 7B, where exploratory movements following the death of the pack’s alpha female are readily apparent in the northern portion of movements of one group.

Both variables describing human land use had strong effects on the probabilities of typical vs. fast movement. Outside the National Park in the GMAs (where prey density is known to be lower^63,66^), wild dogs were significantly more likely to adopt the fast movements of State 3 (Figs. 6 and 7C). Despite this increase in speed within GMAs, wild dogs were significantly less likely to adopt the fast movements of State 3 in areas with high HFI values (Fig. 6), even though GMAs had a higher mean HFI than the National Park (Fig. 3).

Overall, HMMs revealed that (after controlling for seasonal changes associated with denning) wild dogs were more likely to adopt a state of fast, directed movement when outside of the National Park, but shifted to a slower state of movement in areas with a stronger human footprint.

## Discussion

Hidden Markov models revealed strong effects of protection and the human footprint on the movements of African wild dogs. Wild dogs were two times more likely to adopt high speed movement when they were outside the National Park, but they were four times less likely to adopt high speed movement when they were in areas with the highest local values of the Human Footprint Index, relative to areas with the lowest local values.

Faster movements in GMAs relative to the National Park probably have at least two causes. First, the densities of large herbivores are lower in the GMAs^62^, including impala and puku, which are most important prey of African wild dogs in the Luangwa Valley^66^. Lower prey density would be expected to translate into increased effort to locate and kill prey. Second, dispersal and exploratory forays that precede dispersal occurred both in the National Park and the GMAs, but fast exploratory movements to the edge of settled home ranges often included forays into the GMAs and beyond, including movements that reached other National Parks (Luambe, Lukusuzi and North Luangwa) and one movement (not analyzed here because sampling was not sufficiently fine scaled) of more than 350 km in which a wild dog was killed (snared in an agricultural area) just before reaching Lower Zambezi National Park on the border with Zimbabwe.

Regardless of the cause of faster movements in the GMAs, our results also show a clear reduction in high speed movement (paired with little change in turning angles) in areas with a stronger human footprint. This result indicates that human modification of the landscape is likely to reduce connectivity for African wild dogs, despite their prodigious capacity for rapid long distance movement^43^. Despite this effect, observed movements linked wild dogs in South Luangwa with several other National Parks, confirming that wild dogs remain capable of dispersal linking protected areas in Zambia, as in other parts of the continent^43,67^.

Spatial Principal Components Analysis on SNP genotypes from several ecosystems (including the Luangwa population studied here) showed that wild dogs have much less genetic differentiation between ecosystems than sympatric lion populations^22^. This suggests that, although humans do affect wild dog movements, the effect is weak enough (relative to their naturally high baseline level of connectivity) that they remain well-connected. This in turn suggests that efforts to maintain natural connectivity at large scales continue to have reasonable odds of success, providing for recolonization of areas where they have been locally extirpated^36,67^ and maintaining evolutionary processes^68^. For example, the movements described here connected several of the national parks in the Malawi–Zambia Transfrontier Conservation Area, confirming that movements between protected areas at the scale required for TFCAs to function remain possible for wild dogs (which are a focal species for the Africa’s largest TFCA, the Kavango–Zambezi ^57^). However, it also remains possible that the negative effect of humans on wild dog movement seen here is strong enough to reduce connectivity, and that observed genetic patterns are a signature of past movements that are no longer sufficiently common to provide the gene flow that produced them.

Strong tests of the CMC and CDC hypotheses (and broader tests of the relationship between species’ traits and connectivity) will require comparisons across multiple species using standardized methods. To date, most studies have examined single species, with variation between studies in the methods by which connectivity has been assessed, and in the methods by which barriers to movement have been assessed. We used the Human Footprint Index as a measure of anthropogenic resistance to movement because it aggregates a set of variables that are likely to affect the movements of large carnivores^22^, has proven to predict reductions in movement for a wide range of species^51^, and has been extensively validated^49,50^—but reasonable alternatives exist. Human population pressure is one of the eight components of the HFI, and this component is based on resampling of the Gridded Population of the World dataset, available at https://sedac.ciesin.columbia.edu/data/collection/gpw-v4. The HFI human population pressure score converts these population estimates with the equation.

$$pressure score=3.333*\mathrm{log}(density+1)$$

With this conversion, the effect of human density increases asymptotically and saturates at 1000 people/km^2^, which we consider appropriate for large carnivores. Although we considered the HFI to be the most suitable global index of anthropogenic resistance, we compared both the HFI and its human population pressure component to estimates of local population density from the WorldPop project. HFI values did not correlate strongly with the HFI human population pressure component or with WorldPop estimates of human density (Fig. 4), confirming that the HFI is strongly influenced by its other components (as is intended by design). However, the human population component of the HFI and WorldPop estimates of human density were also not tightly correlated, suggesting that differences in the methods used to disaggregate human census data into fine-scaled maps lead to differences in point estimates of human density (at the scale of the movement examined here) that are not intended by design.

With regard to immediate conservation and management, our results show that areas with Human Footprint Index values up to 16 are permeable to wild dogs, although their movements are slowed by higher values within this range. Because the HFI is mapped at one km^2^ resolution across all of Africa, these values can be used to evaluate and prioritize corridors potentially connecting protected populations, and to evaluate the likelihood of natural recolonization events. Many areas linking protected populations of wild dogs have HFI values in this range. For example, a small population of wild dogs in Zambia’s Liuwa Plain National Park was locally extirpated in 2014, coincident with rabies outbreaks in the domestic dog population in and around the park^69^. However, rabies vaccination programs in development for domestic dogs in the area, and HFI values between Greater Liuwa and wild dog populations in Angola’s Mussuma-Cameia area and Zambia’s Kafue National Park, suggest that natural recolonization remains possible if local conditions become favorable in regions of the Liuwa–Mussuma TFCA spanning the border of Zambia and Angola.

In studies of anthropogenic effects on connectivity, it is common to restrict the analysis to a subset of movement data, focusing only on dispersers^32,43^. The strength of this approach is that it focuses on the largest movements. Its corresponding weakness is that it does not directly account for the frequency of such movements, and comparisons between studies are complicated by differences in the definitions of dispersal that are used to restrict the data. Here, by analyzing all observed movements (including, but not restricted to, movements during dispersal), the effects on connectivity of both the magnitude and the frequency of long-distance movements are incorporated, as they should be.

To better resolve differences between species in the effect of humans on connectivity, we need tests of human effects on movement (or current gene flow) and genetic distance for a broader set of carnivores and ecosystems, using standardized methods that allow comparison of the slopes of relationships like those shown in Fig. 1. The Human Footprint Index allows standardization of the measure of human impact used as an independent variable, and has proven utility to detect effects on movement and genetic distance. Hidden Markov models (and their recent extensions that allow for irregular time intervals between locations ^70^) provide a powerful and easily implemented method to model effects on movement. Studies to date are consistent with the Competition—Movement—Connection hypothesis that adaptations allowing wild dogs to range widely while avoiding dominant competitors also allow them to range widely on landscapes affected by human activities. However, our results also show that the human footprint reduces their speed of movement, and stronger tests of the CMC hypothesis await direct, quantitative comparison of the relationships in Fig. 1 for multiple species. As GPS data accumulate from field studies, such tests will provide a better understanding of the traits that cause differences between species in connectivity, rather than simply describing those differences.

## Supplementary information

Supplementary file1
