# Supplementary material for: Hidden Markov Models reveal a clear human footprint on the movements of highly mobile African wild dogs
Source: Sci Rep. 2020 Oct 21;10:17908. doi: 10.1038/s41598-020-74329-w (PMC7578658; doi:10.1038/s41598-020-74329-w)

Supplementary Material for

Hidden Markov Models reveal a clear human footprint on the movements of highly mobile African wild dogs

Scott Creel[1,2,3,*], Johnathan Merkle[1,2], Thandiwe Mweetwa[1], Matthew S. Becker[1,2] Henry Mwape[1], Twakundine Simpamba[4] & Chuma Simukonda[4]

[1] Zambian Carnivore Programme, P.O. Box 80, Mfuwe, Eastern Province, Zambia

[2] Department of Ecology, Montana State University, Bozeman, Montana 59717 USA

[3] Institut för Vilt, Fisk och Miljö, Sveriges lantbruksuniversitet, Umeå, Sweden

4 Zambia Department of National Parks and Wildlife, Chilanga, Zambia

Figure S1. Diagnostic plots of pseudo-residuals and autocorrelation functions for step lengths (left column) and turning angles (right column) from a three-state Hidden Markov Model fit to movements of African wild dogs

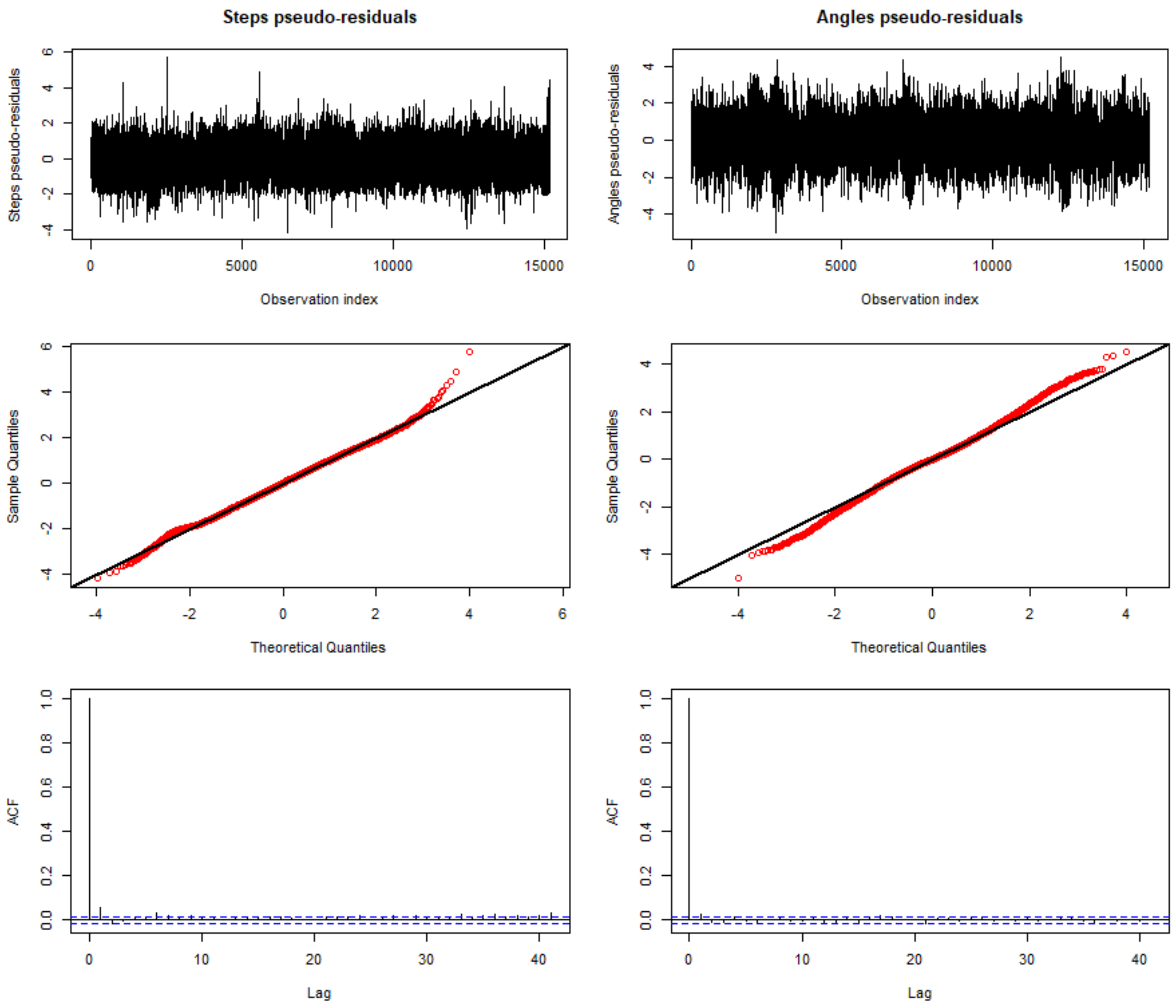

Supplement: Supplementary file 1 — Supplementary file1 [file 41598_2020_74329_MOESM1_ESM.pdf]
